# Whole genome sequencing and the lignocellulose degradation potential of *Bacillus subtilis* RLI2019 isolated from the intestine of termites

**DOI:** 10.1186/s13068-023-02375-3

**Published:** 2023-08-19

**Authors:** Gongwei Liu, Ke Zhang, Hanxuan Gong, Kaiyao Yang, Xiaoyu Wang, Guangchen Zhou, Wenyuan Cui, Yulin Chen, Yuxin Yang

**Affiliations:** 1 College of Animal Science and Technology, Northwest A&F University, Yangling, 712100 Shaanxi China; 2 Qinling Giant Panda Breeding Research Center, Shaanxi Academy of Forestry Sciences, Zhouzhi, 710402 Shaanxi China

**Keywords:** *Bacillus subtilis*, Cellulase, Xylanase, Whole genome sequencing, Lignocellulose degradation, Wheat straw

## Abstract

**Background:**

Lignocellulosic biomass is the most abundant and renewable terrestrial raw material for conversion into bioproducts and biofuels. However, the low utilization efficiency of lignocellulose causes environmental pollution and resource waste, which limits the large-scale application of bioconversion. The degradation of lignocellulose by microorganisms is an efficient and cost-effective way to overcome the challenge of utilizing plant biomass resources. This work aimed to screen valuable cellulolytic bacteria, explore its molecular mechanism from genomic insights, and investigate the ability of the strain to biodegrade wheat straw.

**Results:**

*Bacillus subtilis* (*B. subtilis*) RLI2019 was isolated from the intestine of *Reticulitermes labralis*. The strain showed comprehensive enzyme activities related to lignocellulose degradation, which were estimated as 4.06, 1.97, 4.12, 0.74, and 17.61 U/mL for endoglucanase, β-glucosidase, PASC enzyme, filter paper enzyme, and xylanase, respectively. Whole genome sequencing was performed to better understand the genetic mechanism of cellulose degradation. The genome size of *B. subtilis* RLI2019 was 4,195,306 bp with an average GC content of 43.54%, and the sequence characteristics illustrated an extremely high probability (99.41%) as a probiotic. The genome contained 4,381 protein coding genes with an average GC content of 44.20%, of which 145 genes were classified into six carbohydrate-active enzyme (CAZyme) families and 57 subfamilies. Eight cellulose metabolism enzyme-related genes and nine hemicellulose metabolism enzyme-related genes were annotated by the CAZyme database. The starch and sucrose metabolic pathway (ko00500) was the most enriched with 46 genes in carbohydrate metabolism. *B. subtilis* RLI2019 was co-cultured with wheat straw for 7 days of fermentation, the contents of neutral detergent fiber, acid detergent fiber, hemicellulose, and lignin were significantly reduced by 5.8%, 10.3%, 1.0%, and 4.7%, respectively. Moreover, the wheat straw substrate exhibited 664.9 μg/mL of reducing sugars, 1.22 U/mL and 6.68 U/mL of endoglucanase and xylanase activities, respectively. Furthermore, the fiber structures were effectively disrupted, and the cellulose crystallinity was significantly reduced from 40.2% to 36.9%.

**Conclusions:**

The complex diversity of CAZyme composition mainly contributed to the strong cellulolytic attribute of *B. subtilis* RLI2019. These findings suggest that *B. subtilis* RLI2019 has favorable potential for biodegradation applications, thus it can be regarded as a promising candidate bacterium for lignocellulosic biomass degradation.

**Supplementary Information:**

The online version contains supplementary material available at 10.1186/s13068-023-02375-3.

## Background

Lignocellulose biomass, the most abundant renewable and sustainable carbohydrate source on earth, is very hard to degrade because of the complex crystal structure formed by cellulose, hemicellulose, and lignin. More attention to the low utilization efficiency of lignocellulose results in resource waste, economic loss, and environmental pollution, providing strong impetus to develop pretreatment technologies, commonly including physical, chemical, and biological methods [1]. Compared to physical and chemical approaches, biological pretreatment relying on enzymatic hydrolysis and microbial degradation has obvious advantages, including economy, safety, high performance, and environmental friendliness [2]. Cellulase and hemicellulase can efficiently hydrolyze the glycosidic bond of lignocellulose, which is accomplished through the synergistic effects of multi-enzyme systems, including endoglucanase (EC 3.2.1.4), exoglucanase (EC 3.2.1.91), β-glucosidase (EC 3.2.1.21), and xylanase (EC 3.2.1.8), etc. [3–6]. Initially, endoglucanase disrupts the β-1,4-glycosidic bond in the cellulose macromolecule to reduce the degree of polymerization. Following, exoglucanase acts on the non-reducing end of the crystalline cellulose to obtain soluble non-crystalline cellulose. Ultimately, β-glucosidase degrades oligosaccharide to the final product of glucose [3, 4]. Xylanase primarily recognizes and binds to xylan, cleaving its internal glycosidic bonds to break down polysaccharides into small oligosaccharides and monosaccharides [5, 6]. Microorganisms have a reputation as cell factories for cellulase and xylanase production, although the lignocellulose degradation abilities of microbes such as *Penicillium*, *Aspergillus*, *Bacillus*, *Paenibacillus*, and *Cellulomonas* have been widely studied [3, 7–9]. Continuous and extensive screening of potential cellulolytic bacteria to overcome the challenges associated with industrial production is still attractive.

The termite, which feeds on woody plants, is a representative species of phytophagous insects in nature. The gut symbiotic microorganisms of termites can rapidly digest 74–99% of cellulose and 65–87% of hemicellulose in wood [10]. This valuable characteristic attracted researchers to find excellent bacteria that effectively degrade cellulose in the intestinal tract of termite, including a large number of *Bacillus* spp., *Cellulosimicrobium variable* sp., *Paenibacillus lactis*, *Lysinibacillus macrolides*, *Stenotrophomonas maltophilia*, and other anaerobic microbial communities [11–14]. Although some individual strains have been isolated, studies on applying these natural cellulolytic bacteria in lignocellulose pretreatment are still scarce. In contrast, several genes from the isolates with cellulose degradation functions have been characterized and optimized [15–18]. However, the molecular mechanism of cellulose utilization by microbes from genomic perspectives has not been entirely investigated. Therefore, the profound information on microbial whole genome sequence is necessary for analyzing enzyme genes and metabolic pathways involved in cellulose degradation, which will help to understand the genetic basis and further promote production applications.

In the present study, *Bacillus subtilis* (*B. subtilis*) RLI2019, a dominant cellulase-secreting strain, was isolated from the intestinal of the *Reticulitermes labralis* (a lower termite), and its enzymatic properties were determined. Furthermore, whole genome sequencing of the strain was performed to explore the genetic foundation of cellulolytic activities. Finally, the strain was co-cultured with wheat straw to observe the degradation effect, providing valuable practice for applying in biodegrading lignocellulosic biomass.

## Results and discussion

### Screening and molecular identification of dominant cellulolytic bacteria

**Fig. 1:**
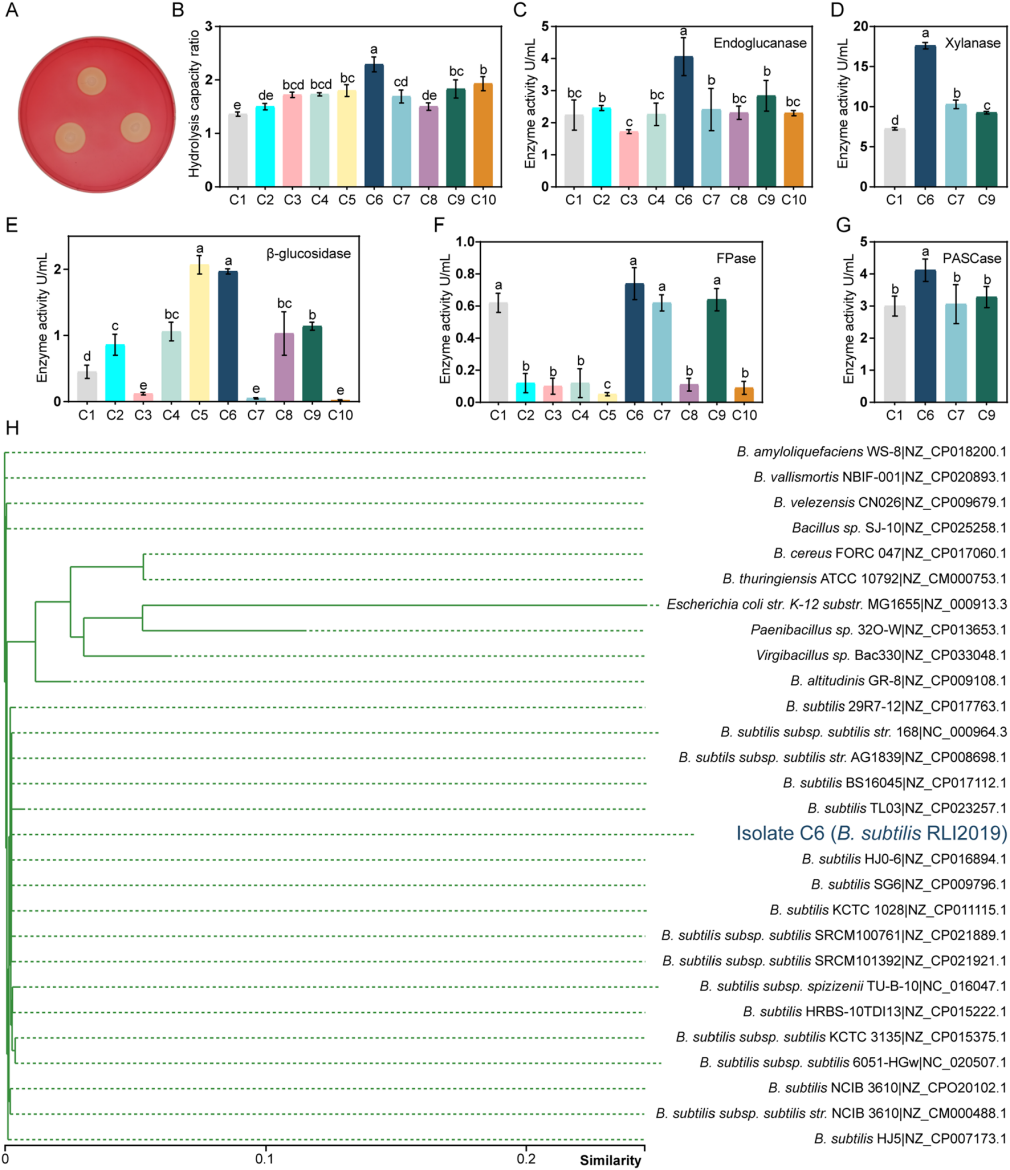
Isolation, identification, and enzyme activities of *B. subtilis* RLI2019. **A** CMC-Na plate with Congo red stain; **B** hydrolysis capacity ratio (HCR): the ratio of hydrolysis zone (D) to colony diameter (d); **C** endoglucanase activity; **D** xylanase activity; **E** β-glucosidase activity; **F** filter paper enzyme activity (FPase); **G** enzyme activity based on PASC substrate (PASCase); **H** *16S rRNA* phylogenetic tree. The different lowercase letters indicate significant differences (*p* < 0.05); all analyses are performed in triplicate (*n* = 3)

Uniform screening procedures were performed to obtain the dominant cellulase-producing strain. Congo red plate staining reflected that clear hydrolysis circles were observed around Isolate C1 to C10, which indicated all strains could degrade CMC-Na. The qualitative assay for CMC-Na hydrolysis showed that Isolate C6 had the highest hydrolysis capacity ratio (HCR: 2.29) compared to other strains (Fig. 1A and B). Many previous studies have isolated cellulolytic bacteria based on HCR screening, such as *Bacillus velezensis* LC1 from *Cyrtotrachelus buqueti* [7], *Paenibacillus lautus* BHU3 from a landfill site [8], and *B. subtilis* from coffee residues [19].

To confirm the cellulolytic ability of all strains, the activities of endoglucanase, β-glucosidase, and FPase were determined. The results showed that Isolate C6 had maximum endoglucanase (4.06 U/mL) (Fig. 1C), β-glucosidase (1.97 U/mL) (Fig. 1E), and FPase (0.74 U/mL) (Fig. 1F). Moreover, Isolates C1, C6, C7, and C9 with higher enzyme activities were selected for estimating the activities of xylanase and PASCase. Similarly, Isolate C6 had the highest xylanase activity (17.61 U/mL) (Fig. 1D) and PASCase (4.12 U/mL) (Fig. 1G) compared with other strains. Notably, the results of enzyme activities and HCR could corroborate each other. For the molecular identification of Isolate C6, the *16S rRNA* sequence similarity comparison in both the NCBI database (Additional file 1: Table S1) and the GTDB database (Additional file 1: Table S2) proved with over 99.8% probability that the strain was *B. subtilis*. Furthermore, the *16S rRNA* phylogenetic tree also indicated that Isolate C6 was closely related to other species or subspecies of *B. subtilis* (Fig. 1H). Taking into account the uniqueness of the strain nomenclature and the characteristics of the isolate, we finally named Isolate C6 as *B. subtilis* RLI2019, which emphasizes that the strain was isolated from the intestine of *Reticulitermes labralis* in 2019. And the designation *B. subtilis* RLI2019 was used throughout the following descriptions.

*Reticulitermes labralis*, one of wood-feeding lower termites, is an effective natural lignocellulose decomposer [20]. Complex microbial composition is critical to the digestive function of the gut system of termites, which enables termites to degrade lignocellulose biomass [10–12]. In the hindgut of lower termite, it has been demonstrated that the flagellates produce endoglucanase, exoglucanase, and β-glucosidase, as well as a multitude of other glycoside hydrolases that are necessary for the digestion of hemicellulose [10]. The bacterial symbionts in the termite gut have been categorized into various phyla, including Bacteroidetes, Fibrobacteres, Spirochaetes, Firmicutes, Actinobacteria, Proteobacteria, and Elusimicrobia, especially *Paenibacillus* and *Bacillus* species are commonly documented as symbionts [14]. Ali et al. [11] isolated five cellulolytic strains from the gut of *Psammotermes hypostoma*, which were identified as *Paenibacillus lactis*, *Lysinibacillus macrolides*, *Stenotrophomonas maltophilia*, *Lysinibacillus fusiformis*, and *B. cereus*. In another study, *B. licheniformis*, *Ochrobactrum intermedium*, and *Microbacterium paludicola* were isolated from the gut of *Microcerotermes diversus* [12]. In our previous study, four strains with cellulose degrading ability were obtained from the gut of *Reticulitermes chinensis*, which were identified as *B. amyloliquefaciens*, *B. cereus*, *B. subtilis*, and *Enterobacter asburiae* [21]. Moreover, the *B. subtilis* strain showed the highest cellulase activity. Considering that *B. subtilis* RLI2019 was isolated from the gut of *R. labralis*, it is unsurprising that the strain showed comprehensive cellulase activities and was considered as a dominant cellulolytic bacterium for further study.

### Enzyme activity characteristics of *B. subtilis* RLI2019

#### Optimization of culture conditions for enzyme production

**Fig. 2:**
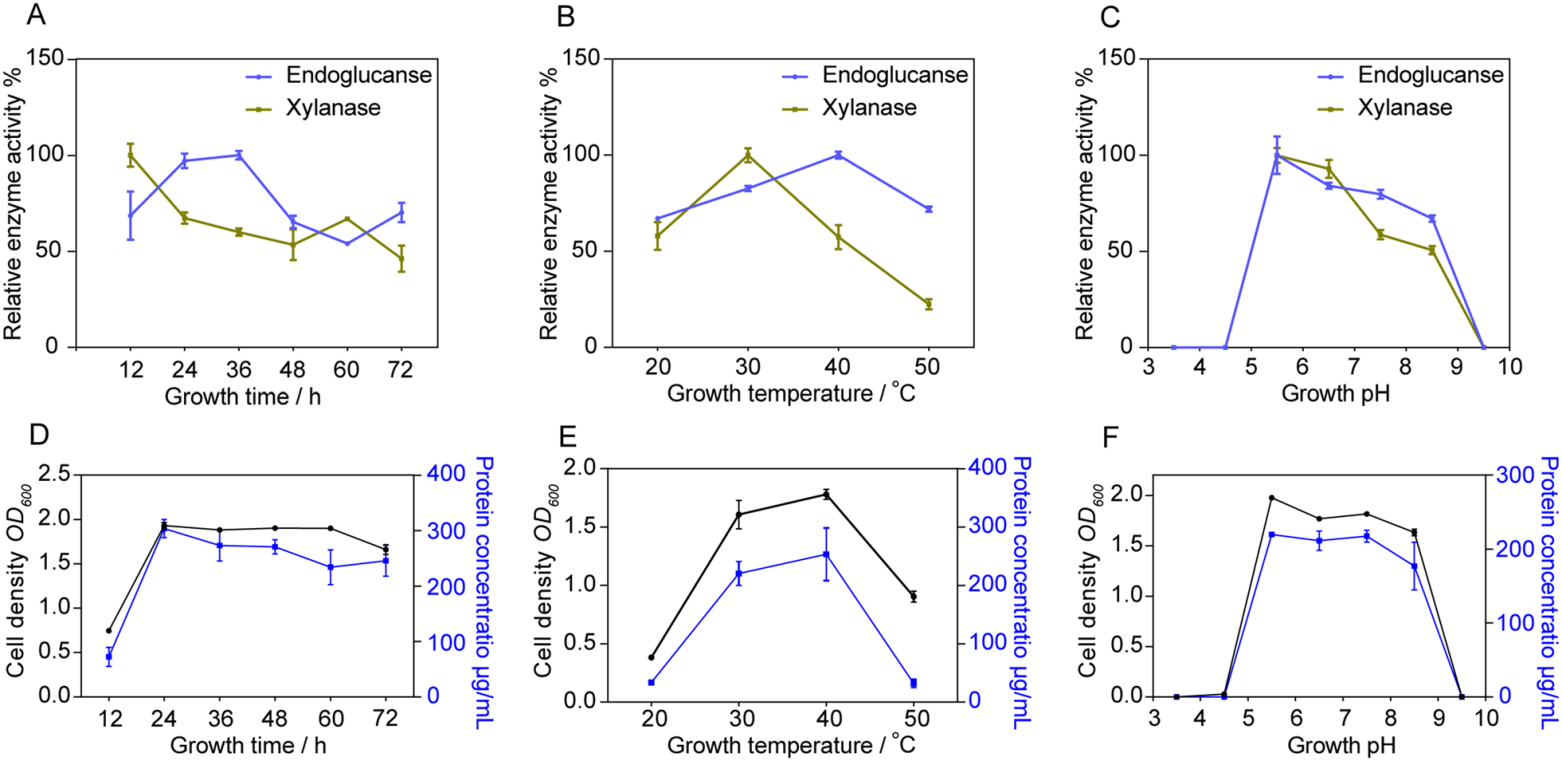
Optimization of cultural conditions for enzyme production in *B. subtilis* RLI2019. **A** Effect of culture time on enzyme activity; **B** effect of culture temperature on enzyme activity; **C** effect of culture pH on enzyme activity. Measurement of cell density and total protein concentration under different culture time (**D**), temperature (**E**), and pH (**F**) conditions. All analyses are performed in triplicate (*n* = 3)

Growth stage, temperature, pH and medium composition could directly affect the ultimate production of extracellular cellulase in microorganisms [18, 22]. Herein, endoglucanase and xylanase activities were determined under different growth conditions, confirming the optimal growth conditions for *B. subtilis* RLI2019 to secrete enzymes. For the effect of growth time, the activities of endoglucanase at 24 h and 36 h were significantly higher than in other periods (Fig. 2A). The characteristics of enzyme protein expression in microorganisms are strictly associated with growth adaptation [23]. We quantified the cell density and total protein concentration at different time points (Fig. 2D), which evidenced continuous cell growth and biomass accumulation so that the amount of enzyme protein increased correspondingly between 12 and 24 h. Although the cell density remained stable after 36 h (Fig. 2D), the endoglucanase activity began to decline probably due to other factors such as accumulation of waste products and partial degradation of the enzyme protein over time [24]. The maximum activity of endoglucanase in *B. subtilis* AS3 was observed between 12 and 36 h [22], our findings corroborated that opinion. However, we observed that xylanase activity was highest at 12 h and then decreased (Fig. 2A). One possible explanation is that endoglucanase hydrolyzed the CMC-Na substrate to release glucose, and the accumulation of glucose resulted in an inhibitory effect on xylanase activity [5, 6].

The appropriate temperature is necessary for growth and enzyme protein secretion. The maximum activities of endoglucanase and xylanase were observed at culture temperatures of 40 °C and 30 °C (Fig. 2B), respectively. At 20 °C and 50 °C, both enzyme activities were significantly decreased, indicating that neither high nor low culture temperatures were conducive to the secretion of enzymes in *B. subtilis* RLI2019. A previous study also found maximum endoglucanase activity at 40 °C by *B. subtilis* MUS1, and the activities decreased at 30 °C followed by 50 °C [25]. Similarly, some studies reported that the highest xylanase activities of *Bacillus* were observed at growth temperatures between 30 °C and 35 °C [6, 24, 26, 27]. Variations in growth temperature caused differences in enzyme activities, which may be related to the fact that temperature alters the growth rate and extracellular enzyme secretion. *B. subtilis* RLI2019 growth between 30 °C and 40 °C maintained relatively high biomass accumulation and total protein concentration, while at 20 °C and 50 °C did not grow well thus resulting in decreased enzyme production (Fig. 2E). Additionally, it is indeed an interesting phenotype that endoglucanase and xylanase have different optimal temperatures. However, the exact molecular mechanisms of this difference are unclear based on the current limited knowledge. It might be an exciting direction to explain it for future studies by exploring the specific secretion process of the enzymes.

After culture of *B. subtilis* RLI2019 in a different medium (pH from 3.5 to 9.5) for 24 h, the activities of endoglucanase and xylanase were measured (Fig. 2C). Both maximum activities were observed at pH 5.5 and then decreased with increasing pH, while minimum relative enzyme activities were achieved at pH 8.5. In contrast, the enzyme activities could not be detected at pH 3.5, 4.5, and 9.5 (Fig. 2C). The effect of medium pH on enzyme activity is mainly related to the growth rate of microorganisms was changed by pH [23]. Strongly acidic and strongly alkaline environments inhibit growth and biomass accumulation. These results were confirmed by the maximum cell density and total protein concentration at pH 5.5, while these indexes could not be detected at pH 3.5, 4.5, and 9.5 (Fig. 2F). A study by Malik et al. [23] supported this notion, as a strong positive correlation was observed between the cell density and cellulase production of *B. subtilis* CD001, with increasing medium pH from 4.0 to 7.0, the cell mass also increased correspondingly, resulting in increased enzyme activity. It is common for isolates from different environments to exhibit variations in growth pH preference. Siu-Rodas et al. [19] isolated *B. subtilis* from coffee grounds at different pH composting environments, and the isolates exhibited cellulolytic activity under pH 4.8 or pH 9.3 conditions. During the process of submerge fermentation, *B. subtilis* BS04 [26] and *B. subtilis* JJBS250 [27] obtained the highest xylanase production at pH 8.0 and pH 4.0, respectively. Wang et al. [28] suggested that *B. subtilis* isolated from herb compost had the maximum cellulase activity in the culture medium with an initial pH 6.5. Brune's [10] study emphasized that the whole intestine of lower termites was neutral and weakly acidic (pH 5.0–7.0). In the present research, *B. subtilis* RLI2019 was isolated from the lower termite gut. It is reasonable to infer that the unique gut environment of termites significantly contributed to the growth pH preferability of the strain.

#### Optimization of reaction conditions for enzyme activities

**Fig. 3:**
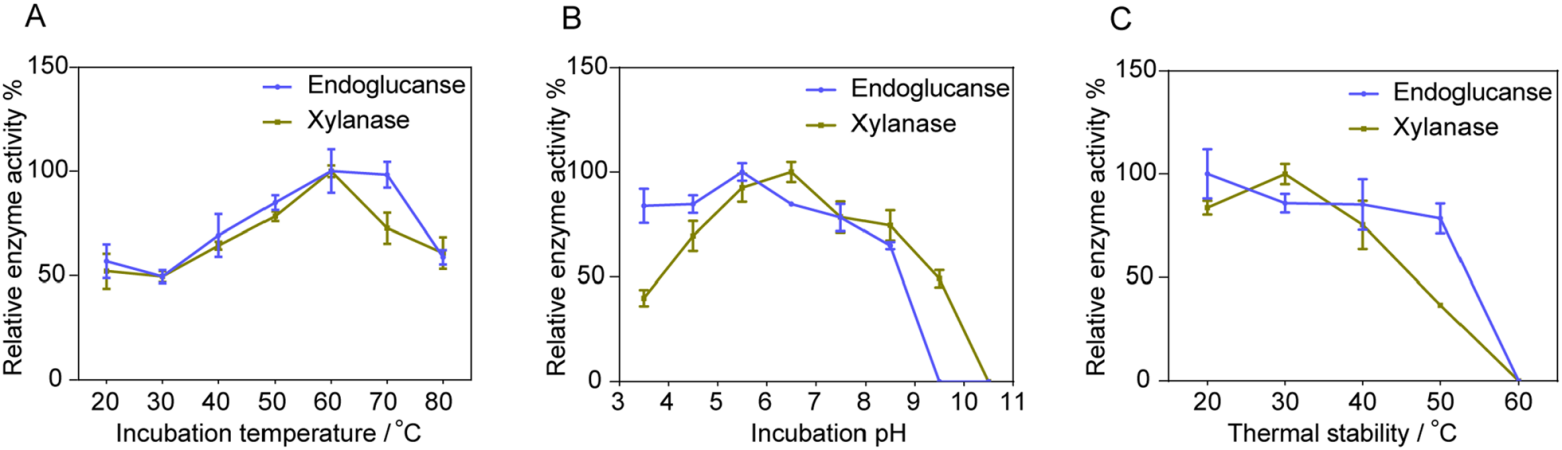
Optimization of reaction conditions for enzyme activities in *B. subtilis* RLI2019. **A** detection of optimum incubation temperature for enzyme activity; **B** detection of optimum incubation pH for enzyme activity; **C** detection of thermal stability. All analyses are performed in triplicate (*n* = 3)

The crude enzyme solution was collected under optimal culture conditions, i.e., culture medium pH 5.5, with incubation at 37 °C for 24 h. The effect of reaction temperature on relative enzyme activities was studied by assaying the enzyme activity at a wide range of temperatures (20–80 °C). The optimal incubation temperature of endoglucanase and xylanase in *B. subtilis* RLI2019 was 60 °C (Fig. 3A). This finding was consistent with previous studies, which suggested that the most favorable reaction temperature of cellulase enzyme was 60 °C in *B. subtilis* [18, 19]. Surprisingly, the relative enzyme activities of endoglucanase and xylanase at 70 °C were 98.3% and 72.7%, respectively (Fig. 3A). Enzymatic reaction rate is limited at low temperatures, meanwhile extremely high temperature makes the enzymes to be partially or completely inactive, both of which can destabilize the stability of enzymatic reaction [3, 18, 23]. Previous studies have shown that the optimum reaction temperature for cellulase was 40–50 °C [9, 22, 28]. Overall, enzyme activities of *B. subtilis* RLI2019 showed great adaptability under high temperatures, which is important for industrial applications in hot processing.

Relative enzyme activities of endoglucanase were maintained over 78.0% when the reaction pH was between 3.5 and 7.5. The optimal reaction pH of endoglucanase was pH 5.5 (Fig. 3B). On the other hand, for the activity of xylanase, the optimal reaction pH was between 5.5 and 6.5; when the pH was set as 7.5, 8.5, and 9.5, the relative enzyme activities were 78.5%, 74.5%, and 49.2%, respectively (Fig. 3B). Interestingly, the optimal reaction pH (Fig. 3B) was similar to the optimal growth pH (Fig. 2C), these findings supported the positive adaptation of *B. subtilis* RLI2019 to neutral and weakly acidic environments. Reaction pH is a significant avenue affecting cellulase activity, numerous studies have emphasized that cellulase from *B. subtilis* is stable in neutral and weakly acidic conditions. For example, cellulase derived from *B. subtilis* CD001 displays the highest activity at pH 5.0, with more than 85% of the maximum activity observed between pH 4.0 and pH 5.5, while the enzyme activities are minimal outside of this range [18]. Study on the enzymatic properties of cellulase produced by *B. subtilis* isolated from herbaceous compost, it was found that the optimal reaction pH was 5.0, and exhibited 50% relative activity over a pH range of 3.0 to 9.0 [28]. Although the endoglucanases and xylanases of *B. subtilis* RLI2019 have wide adaptability in pH range, the activities at alkaline condition (pH > 9.5) are not ideal. However, in some cases, the highest cellulase activity of *B. subtilis* is determined under alkaline conditions [15, 22]. Compared to our results with these studies, this difference can be attributed to the strain-specific of enzymatic characteristics. Noticeably, although the impact of culture conditions on cellulase activities is caused by differences in enzyme protein secretion (Fig. 2), different reaction temperatures and pH values are more likely to alter the kinetic parameters of the enzyme, including rate constants, inactivation rate constants, and hydrolysis rates [9, 29, 30].

### Thermostability of endoglucanase and xylanase

For endoglucanase, residual enzyme activities were 100%, 85.5%, 85.2%, and 78.5% after incubation at 20, 30, 40, and 50 °C for 1 h. In comparison, the activities of xylanase in the same conditions showed 83.6%, 100%, 75.4%, and 36.8%, respectively (Fig. 3C). Notably, the activities of endoglucanase and xylanase could be unmeasured when the incubation temperature exceeded 60 °C, demonstrating that both enzymes are extremely susceptible to inactivation when preserved under high temperature conditions. Thermostability of enzymes depends on protein structural features and the interactions between molecular covalent bonds, which confer a high degree of stability [31]. Enzymes are usually more stable at lower temperatures due to the reduced thermal motion of molecules at lower temperatures. Generally, the thermally stable of cellulase produced by *B. subtilis* is below 80 °C to survive under particular conditions [18, 19, 28]. However, enzymes produced by *B. subtilis* RLI2019 exhibited instability at higher temperatures (> 50 °C), which may be related to long-term acclimation to the lower temperature environment in the intestine of termite. A behavioral survey on various species of termites at different temperatures revealed that the most comfortable living temperature was 30 °C, moreover, all termites died at 40 °C for 5 days [32]. A review highlighted that cold-active cellulases produced by bacteria exhibit higher catalytic activities at lower temperatures (< 40 °C), but become progressively inactivated as the temperature increases, which is attributed to the unfolding and denaturation of the protein structure [33].

### Sequence characteristics of the complete genome

**Fig. 4:**
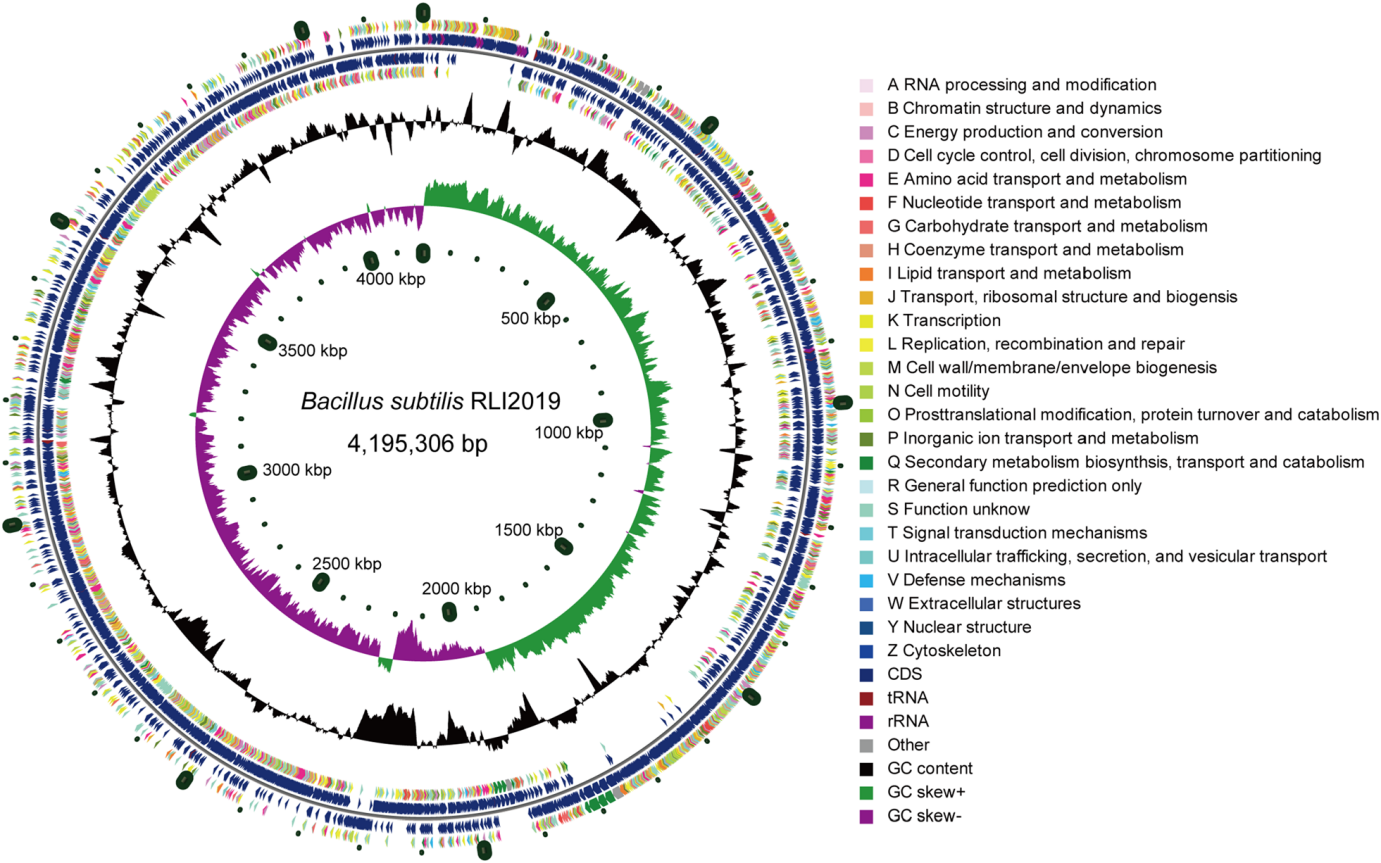
CGView genome map of *B. subtilis* RLI2019. From the outside to the inside, the first and fourth circles are functional COG classifications on the positive and negative chains, respectively; the second and third circles are the coding sequences (CDSs) on the positive and negative chains; the fifth circle is the GC content; the sixth circle is the GC-skew value, and the innermost circle is the genome size indicator

Whole genome sequencing was performed to an average depth of 602.43 × . The de novo assembly found that the genome of *B. subtills* RLI2019 is a single circle chromosome without plasmid; the genome size and total GC contents were 4,195,306 bp and 43.54%, respectively (Table 1). The CGView genome map (Fig. 4) and Circos circle map (Additional file 1: Figure S1) were generated based on complete sequence information. A total length of 3,712,287 bp coding genes were identified with average length (847.36 bp), GC content (44.20%) and the ratio of gene length to the genome (88.49%). Furthermore, 4,381 CDSs were predicted, which were further functionally annotated in NR (4,381), Swiss-Prot (3,999), Pfam (3,603), COGs (3,261), GO (3,145), and KEGG (2,341) database (Table 1). We further performed homologous gene clustering analysis by comparing the genome of *B. subtilis* RLI2019 with other reported cellulose-degrading *B. subtilis* strains. The results showed that these strains shared a core gene set of 3,651 genes. The number of unique genes for *B. subtilis* RLI2019, *B. subtilis* TLO3, *B. subtilis* 30VD-1, *B. subtilis* Gd7, and *B. subtilis* CRN1 were 168, 132, 168, 179, and 232, respectively (Additional file 1: Figure S2).

**Table 1:** The basic genome features of *B. subtilis* RLI2019

Features	Results	Features	Results
Genome size (bp)	4,195,306	5S rRNA	10
GC content (%)	43.54	16S rRNA	10
Coding genes	4,381	23S rRNA	10
Gene total length (bp)	3,712,287	tRNA	85
GC content in gene region (%)	44.20	NR annotation	4,381
Coding genes/genome (%)	88.49	Swiss-Prot annotation	3,999
Average gene length (bp)	847.36	Pfam annotation	3,603
Intergenic region length (bp)	483,019	COG annotation	3,261
GC content in intergenic region (%)	38.46	GO annotation	3,145
Intergenic length/genome (%)	11.51	KEGG annotation	2,341

**Fig. 5:**
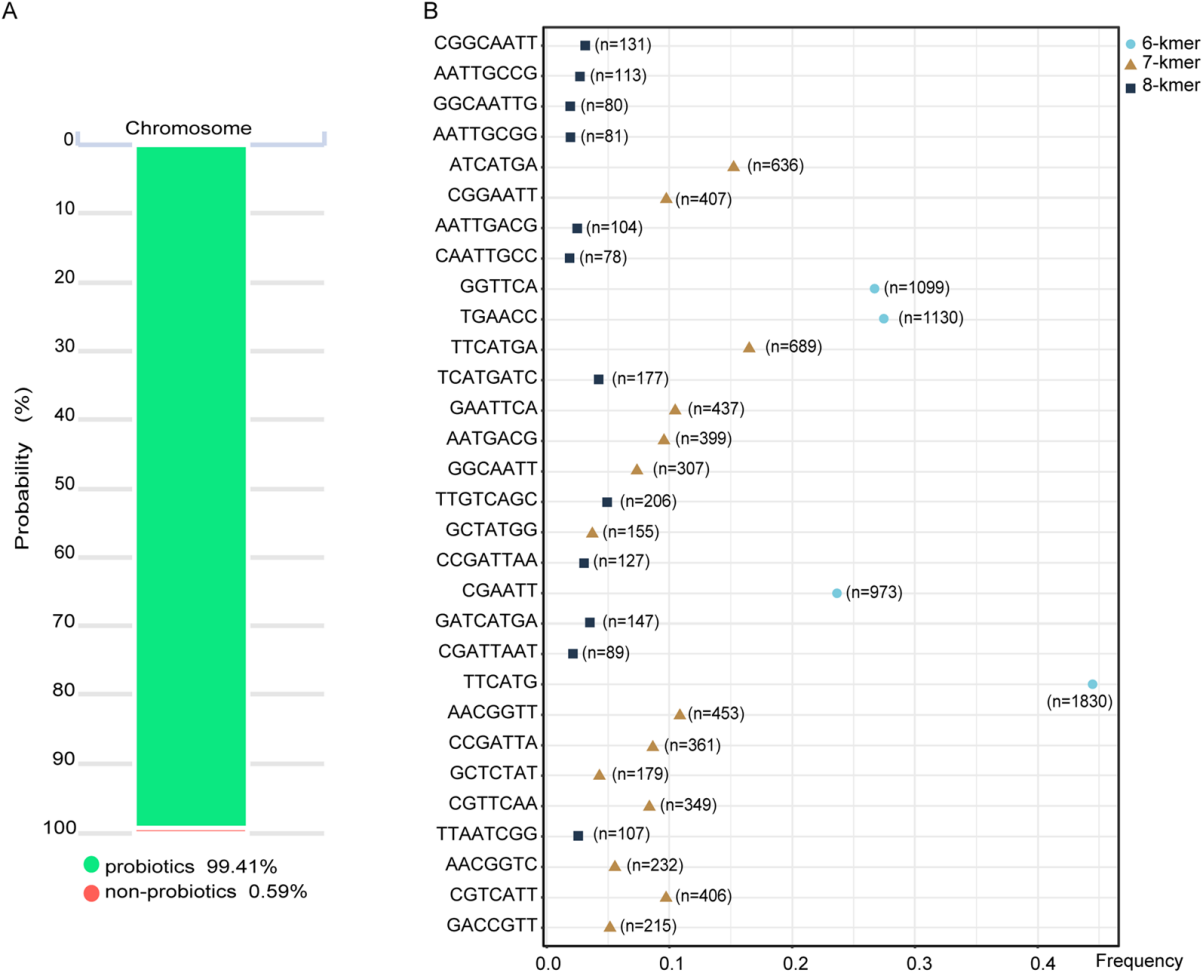
Probiotic characterization of *B. subtilis* RLI2019 genome. **A** Probability prediction of probiotics; **B** sequence analysis of the top 30 core features about probiotics, ranked by importance, with numbers indicating the frequency of each sequence

*B. subtilis* is a generally recognized as safe (GRAS) and commercialized probiotic bacterium, which has been widely used in agriculture, medicine, industry, and manufacturing [34]. Based on this well-known viewpoint, although it is not surprising that *B. subtilis* RLI2019 is also a probiotic, the identification of probiotic was still performed using the iProbiotics web server [35]. The iProbiotics database is a bioinformatics platform that uses machine learning algorithms to predict the possibility of the strain as a probiotic. It achieved this by analyzing the presence of certain kmers, which are short and contiguous sequences of nucleotides in the genome. The prediction tool has been trained on a large dataset of 239 known probiotic strains (41 species) and 412 non-probiotic strains (80 species), enabling it to make accurate predictions based on the presence or absence of specific kmers. Furthermore, the iProbiotics database has summarized 184 core characteristic sequences associated with probiotics, including the top 30 sequences ranked by importance [35]. The predicted results revealed that *B. subtilis* RLI2019 could be almost confirmed (99.41%) as a probiotic (Fig. 5A). The core characteristic sequences of the top 30 most important in probiotics showed enrichment in the genome (Fig. 5B). The highest abundance of 6-kmer (TTCATG), 7-kmer (TTCATGA), and 8-kmer (TTGTCAGC) occurred 1,830, 689, and 206 times, respectively, providing more evidence that *B. subtilis* RLI2019 is a potential probiotic strain. Furthermore, we comparatively analyzed the top 30 sequence frequencies in *B. subtilis* RLI2019 and other four known cellulose-degrading *B. subtilis* (Additional file 1: Table S3). Remarkably, the total frequency of the top 30 sequences was higher in *B. subtilis* RLI2019 compared to *B. subtilis* TLO3, *B. subtilis* 30VD-1, *B. subtilis* Gd7, and *B. subtilis* CRN1 by 5.4%, 12.5%, 8.2%, and 9.4%, respectively. Moreover, almost all of the core characteristic sequences appeared more frequently in *B. subtilis* RLI2019, indicating that *B. subtilis* RLI2019 had the most advantage over other four *B. subtilis* strains in terms of probiotic properties. However, the iProbiotics database has certain limitations in predicting probiotics, such as the limited number of *B. subtilis* included and the exclusion of sequence characteristics from pathogenic *B. subtilis* in the training dataset. Therefore, it is necessary to conduct comprehensive safety assessments in future studies through cytotoxicity assays and animal feeding experiments, to provide more convincing evidence for *B. subtilis* RLI2019 as a probiotic.

### Gene function annotation

#### GO and COG annotation

The number of genes annotated by GO database was 3,145, accounting for 71.79% of all genes, which were classified into three GO categories (Additional file 1: Figure S3A), including biological process (2,406 genes), cellular component (1,832 genes), and molecular function (2,450 genes). Considering the excellent cellulose degradation ability of *B. subtilis* RLI2019, the GO items associated with carbohydrate metabolism were deeply investigated. The results demonstrated that 104 genes are related to carbohydrate metabolism (Additional file 2: Table S4). In total, 3,261 functional genes were annotated based on the COG database, which accounted for 74.44% of all genes (Table 1). A total of 1,277 genes were classified into the metabolism category (Additional file 1: Figure S3B), including 255 genes directly related to carbohydrate transport and metabolism (Additional file 3: Table S5), which may confer *B. subtilis* RLI2019 with a wide variety of cellulolytic components. Herein, the COG categories agreed with the previous studies about cellulolytic bacteria [7, 36]. These highly productive COGs regulated carbohydrate metabolism through a combination of biological processes, such as substrate transport, polysaccharide hydrolysis, and glucose uptake [37, 38].

### KEGG annotation and CAZyme analysis

**Fig. 6:**
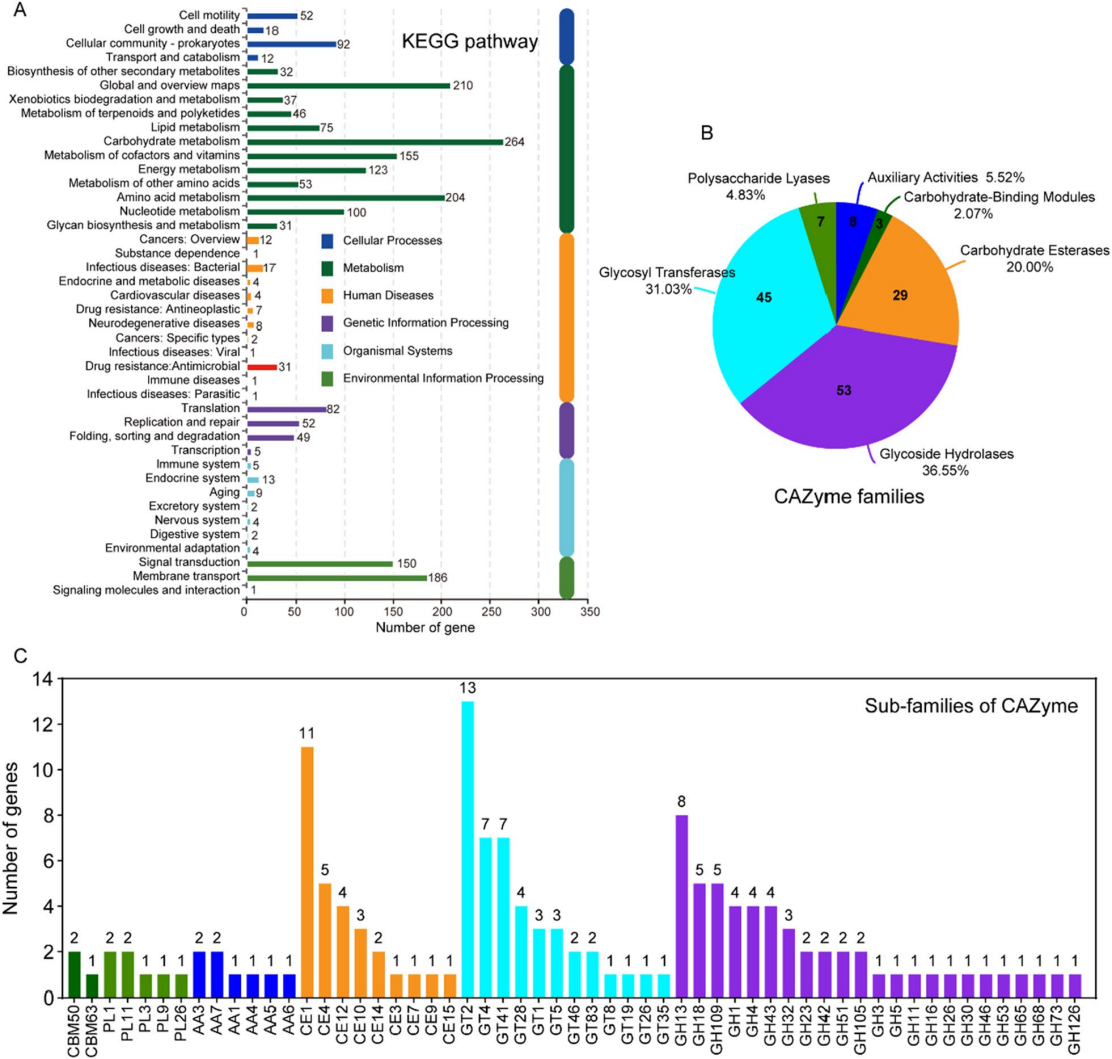
Genome-wide functional annotation of *B. subtilis* RLI2019. **A** KEGG pathway annotation; **B** carbohydrate-active enzyme (CAZyme) annotation; **C** level 2 classification of CAZyme

The genome of *B. subtilis* RLI2019 was divided into six major KEGG categories (Fig. 6A), of which metabolism contained the largest number of genes (1,330). In the level 2 pathways of metabolism, carbohydrate metabolism, global and overview maps, and amino acid metabolism were the top 3 pathways with 264, 210, and 204 genes, respectively (Fig. 6A). As like GO and COG annotations, carbohydrate metabolism was emphasized in the KEGG pathways (Additional file 4: Table S6). The starch and sucrose metabolism pathway (ko00500) is the most enriched with 46 genes and showed similarity to other cellulolytic strains [7, 8, 36]. Many enzymes that promote hydrolysis of lignocellulosic were observed in the ko00500 pathway (Table 2), including 6-phospho-β-glucosidase (EC:3.2.1.86), α-glucosidase (EC:3.2.1.20) and endoglucanase (EC:3.2.1.4).

**Table 2:** Comparative genomic analyses of CAZymes with other *B. subtilis* strains

*B. subtilis* strains	GHs	GTs	CEs	PLs	AAs	CBMs	Total
RLI2019	53	45	29	7	8	3	145
TLO3	54	41	28	7	7	3	140
30VD-1	52	42	29	7	8	3	141
Gd7	52	45	29	7	8	2	143
CRN1	53	44	29	7	7	3	143

To gain insight into the molecular mechanisms of cellulose degradation in *B. subtilis* RLI2019, a summary of CAZyme genes was performed based on the CAZy database. The 145 CAZyme genes were grouped into six families, with the number of genes in GHs, GTs, CEs, AAs, PLs, and CBMs were 53, 45, 29, 8, 7, and 3, respectively (Fig. 6B and Additional file 5: Table S7). Specifically, these genes were classified into 57 subfamilies, GT2 (13 genes), CE1 (11 genes), and GH13 (8 genes) had the most abundance (Fig. 6C). In comparison, the number of CAZyme genes for *B. subtilis* TLO3, *B. subtilis* 30VD-1, *B. subtilis* Gd7, and *B. subtilis* CRN1 were 140, 141, 143, and 143, respectively (Table 2). In the genome of *B. subtilis* TL03, coding genes related to starch and cellulose hydrolysis were detected, making the strain a potential candidate for industrial degradation of lignocellulosic biomass [39]. *B. subtilis* 30VD-1, isolated from the palm rhizosphere, showed strong activities of cellulase, xylanase, pectinase, and chitinase [40]. *B. subtilis* VS15 was isolated from decomposing logs, had the excellent capacity for cellulose degradation. After two rounds of genome shuffling, the mutated strain *B. subtilis* Gd7 was obtained, which further increased its cellulase activity by 167% [41]. *B. subtilis* CRN1 derived from camel rumen showed high cellulase and hemicellulase activities, and was annotated with multiple glycoside hydrolase genes involved in plant biomass deconstruction [42]. Although the CAZyme annotation indicated that *B. subtilis* RLI2019 had slightly more enzymes with carbohydrate-degrading activity, the results clearly demonstrated that these strains shared similar distribution patterns of CAZyme genes, which can partially explain the molecular mechanisms underlying the strong cellulose-degrading ability of these *B. subtilis* strains.

The pathways of cellulose and hemicellulose degradation were analyzed, which identified eight and nine directly related genes, all belonging to the GH families except for gene2072 was defined as CBM (Table 3). GHs, which catalyze the breakdown of glycosidic bonds, are commonly found in the natural environment and can hydrolyze cell wall polysaccharides such as cellulose and hemicellulose [43]. The synergistic action of endoglucanase, exoglucanase, and β-glucosidase constituted the classical mechanism of cellulose degradation [3]. In the genome of *B. subtilis* RLI2019, gene2006 was identified as an endoglucanase (Table 3), which was considered to be the rate-limiting step in cellulose degradation [4]. Moreover, gene3743 was identified as a cellulase (EC: 3.2.1.89) in the Pfam database (Table 3). It is noteworthy that exoglucanase and β-glucosidase were unmatched in the chromosome of *B. subtilis* RLI2019, indicating a defect with an incomplete cellulase system, which is consistent with previous findings in other *Bacillus* species [7, 44]. However, gene4296 was identified as β-glucanase (Table 3), characterized and expressed from *B. subtilis* 1AJ3 with high β-glycoside hydrolase activity in a recent report [17]. Besides, six 6-phospho-beta-glucosidase (EC: 3.2.1.86) genes were annotated (Table 3), which may well explain the detection of β-glucosidase activity in *B. subtilis* RLI2019 (Fig. 1E).

**Table 3:** Annotated genes in cellulose-degrading and hemicellulose-degrading pathways of *B. subtilis* RLI2019

Classification	Gene ID	CAZy class	CAZy family	Function description
Cellulose-related	gene0394	GHs	GH1	6-phospho-beta-glucosidase (EC:3.2.1.86)
	gene0651	GHs	GH1	6-phospho-beta-glucosidase (EC:3.2.1.86)
	gene2006	GHs	GH5_2	endoglucanase (EC:3.2.1.4)
	gene4239	GHs	GH4	6-phospho-beta-glucosidase (EC:3.2.1.86)
	gene4313	GHs	GH1	6-phospho-beta-glucosidase (EC:3.2.1.86)
	gene4403	GHs	GH1	6-phospho-beta-glucosidase (EC:3.2.1.86)
	gene2072	CBMs	CBM63	endoglucanase (NR annotation)
	gene4296	GHs	GH16	beta-glucanase
Hemicellulose-related	gene0655	GHs	GH26	beta-mannosidase (EC:3.2.1.78)
	gene1940	GHs	GH43_11	1,4-beta-xylosidase (EC:3.2.1.37)
	gene2008	GHs	GH30_8	endo-1,4-beta-xylanase (EC:3.2.1.136)
	gene2009	GHs	GH43	arabinoxylan arabinofuranohydrolase (EC:3.2.1.55)
	gene2093	GHs	GH11	endo-1,4-beta-xylanase A (EC:3.2.1.8)
	gene3110	GHs	GH51	alpha-N-arabinofuranosidase (EC:3.2.1.55)
	gene3132	GHs	GH51	alpha-N-arabinofuranosidase (EC:3.2.1.55)
	gene3141	GHs	GH43_5	arabinan endo-1,5-alpha-L-arabinosidase (EC:3.2.1.99)
	gene3743	GHs	GH53	arabinogalactan endo-1,4-beta-galactosidase (EC:3.2.1.89); cellulase (Pfam annotation)

GTs are fundamental to catalyze the transfer of sugar from activated donor sugars to saccharide and non-saccharide acceptors [45]. Non-carbohydrate modifications inhibited the hydrolysis efficiency of polysaccharides by GHs, which could be resolved with the synergistic action of CEs [46]. AAs are catalytic modules involved in plant cell wall degradation and are associated with various CBMs [47]. PLs cleaved glycosidic bonds through a β-bond elimination mechanism rather than a hydrolysis process [48]. CBMs can bind carbohydrate ligands, increasing enzyme affinity to substrates and improving catalytic efficiency [49]. In the *B. subtilis* RLI2019, the subfamilies of GTs, CEs, AAs, PLs, and CBMs were 12, 9, 6, 5, and 2, respectively (Fig. 6C). In summary, the complex diversity of CAZyme types provided genetic evidence for the powerful cellulolytic capacity of *B. subtilis* RLI2019, its comprehensive cellulase system is expected to be achieved in the future based on genetic engineering technology.

### Degradation effect of *B. subtilis* RLI2019 on wheat straw

**Fig. 7:**
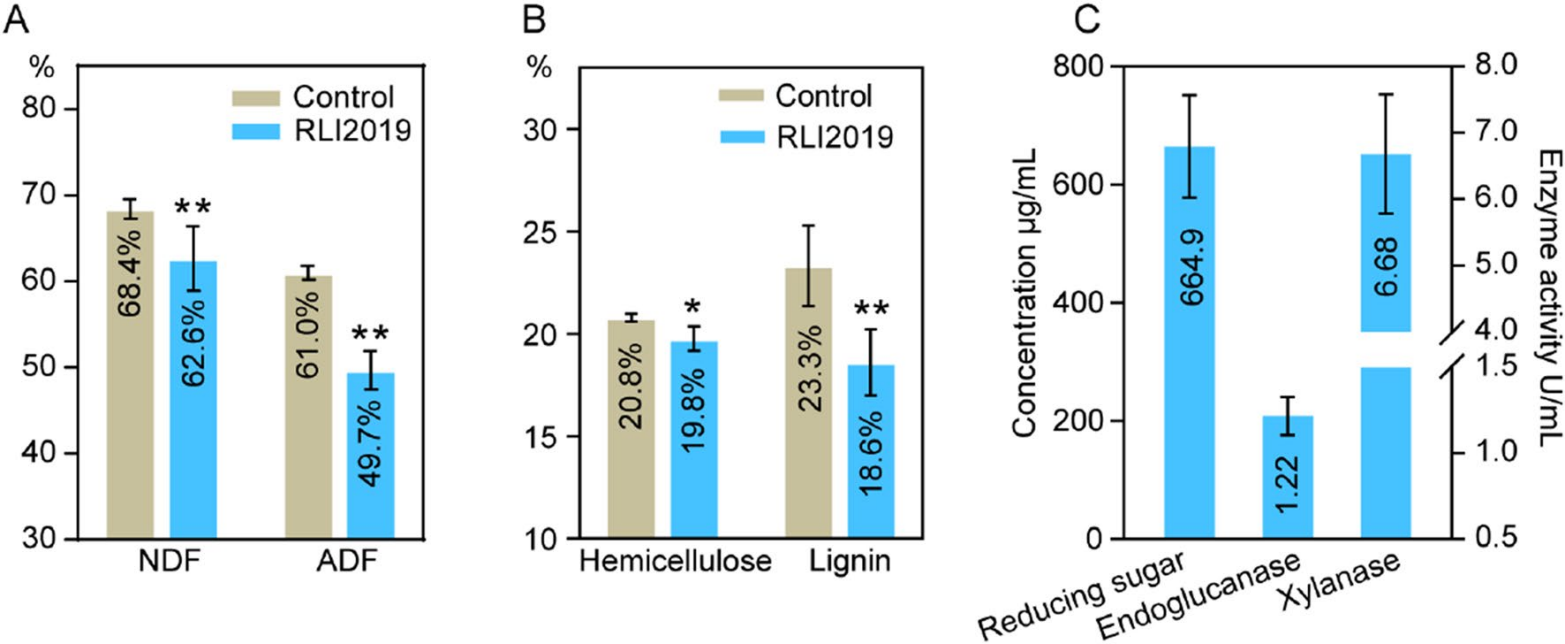
Degradation efficiency of *B. subtilis* RLI2019 on wheat straw. **A** NDF and ADF contents; **B** hemicellulose and lignin contents; **C** reducing sugar content and enzyme activities in wheat straw substrate; Control: the sample of wheat straw without strain treatment; RLI2019: the sample of wheat straw was degraded by *B. subtilis* RLI2019. All groups are performed in triplicate (*n* = 3), * indicates *p* < 0.05, ** indicates *p* < 0.01

Crop straw is mainly composed of cellulose, hemicellulose, and lignin. *B. subtilis* RLI2019 was co-cultured with wheat straw for solid-state fermentation to evaluate its potential application in lignocellulosic degradation. After 7 days fermentation, the NDF content of wheat straw decreased from 68.4% to 62.6%. Meanwhile, the ADF content was reduced from 61.0% to 49.7% (Fig. 7A). Moreover, the contents of hemicellulose and lignin were decreased from 20.8% to 19.8% and 23.3% to 18.6%, respectively (Fig. 7B). Correspondingly, the concentration of glucose in the substrate was 664.9 μg/mL, and the activities of endoglucanase and xylanase were detected as 1.22 U/mL and 6.68 U/mL (Fig. 7C), respectively, indicating that *B. subtilis* RLI2019 was able to degrade lignocellulosic components to produce monosaccharides and maintain growth using wheat straw as a carbon source. The magnitude of reduction in cellulose, hemicellulose, and lignin content can reflect the degradation ability of microorganisms to lignocellulose biomass. Complex *B. subtilis* agents were added to corn silage for fermentation, resulting in a reduction of approximately 3.5% and 5% in the content of NDF and ADF, respectively [50]. An experiment has used *B. licheniformis* isolated from termites to degrade wheat straw, and although ADF digestibility was slightly increased by 2.4%, both NDF and ADF contents were unchanged [12]. Ma et al. [16, 17] found that *B. subtilis* 1AJ3 isolated from rotten wood had limited ability to degrade cellulose, xylan, and acid-insoluble lignin from wheat straw, moreover, only approximately 40 μg/mL of reducing sugars and 0.04 U/mL of endoglucanase activity were detected in the substrate. The endoglucanase activity (1.31 U/mL) was observed in the optimized medium supplemented with 1% wheat straw in *B. subtilis* CD001 isolated from cow dung [18]. The degradation rate of *B. subtilis* RLI2019 on detergent fibers, hemicellulose, and lignin were much higher than previously reported, demonstrating its efficient hydrolysis ability on lignocellulose.

**Fig. 8:**
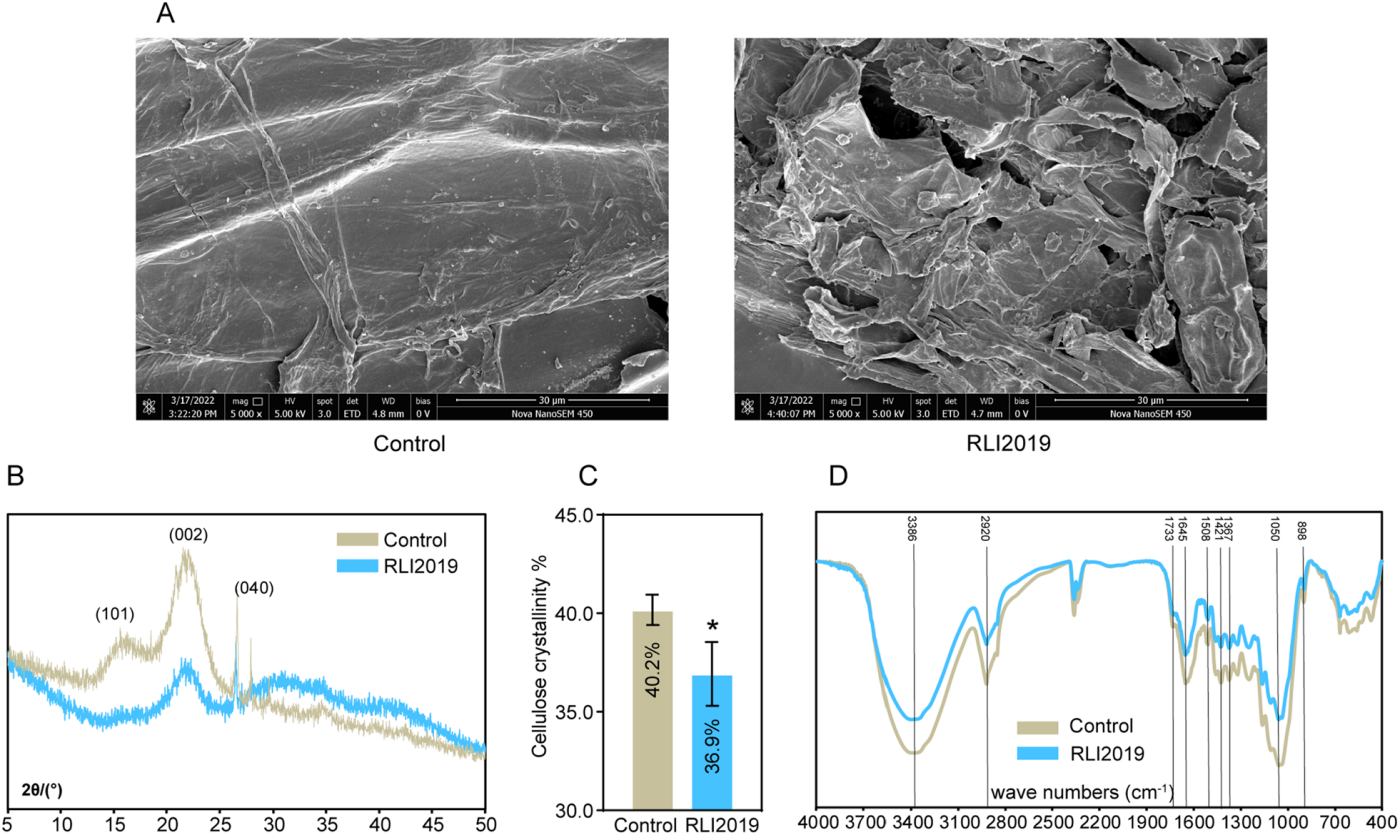
Analyses of wheat straw fiber structure using FE-SEM, XRD, and FTIR. **A** FE-SEM images of surface structure, the images were observed at 5,000 times; **B** XRD spectrum of wheat straw; **C** quantitative analysis of cellulose crystallinity; **D** FTIR spectrum of wheat straw; Control: the sample of wheat straw without strain treatment; RLI2019: the sample of wheat straw was degraded by *B. subtilis* RLI2019. All groups are performed in triplicate (*n* = 3), * indicates *p* < 0.05

The effects of *B. subtilis* RLI2019 degradation on the structure and functional groups of wheat straw were confirmed using FE-SEM, FTIR, and XRD analysis. FE-SEM visualization showed that the surface of degraded wheat straw was rough, loose, and disordered in shape (Fig. 8A). This extensive damage to the fiber structure after *B. subtilis* treatment was also observed in previous studies [17, 18]. The XRD pattern revealed that the main characteristic peaks (101 and 002) were significantly reduced in the sample after fermentation (Fig. 8B). Further calculation of cellulose CrI found that the CrI of wheat straw was decreased from 40.2% to 36.9% (Fig. 8C). The reduction in CrI is an indicator of a reduced cellulose content or a disruption of the fiber structure. Ma et al. [16] reported that *B. methylotrophicus* reduced the CrI of wheat straw merely by 1%. In a previous study, wheat straw was directly saccharified by treatment with crude enzyme solutions produced by *B. subtilis* CD001, which resulted in a 1.39% change in cellulose CrI of the biomass [18]. In sharp contrast, our results reached 3.3%, indicating the high efficiency of *B. subtilis* RLI2019 pretreatment.

FTIR is a powerful technique to identify the structure and composition of various compounds, which can be used to analyze the functional groups of lignocellulose based on different characteristic peaks. The strong peaks around 3,400 cm^−1^ represent hydroxyl (–OH) stretching in cellulose and hemicellulose [18, 51], while around 2,900 cm^−1^ relate to the symmetric stretching vibration of methyl (–CH) and methylene (–CH_2_) groups [52, 53]. The absorption band at 1,728 cm^−1^ is assigned to C = O stretching vibration of hemicellulose, while the peak at 1,508 cm^−1^ is associated with aromatic ring C = C stretching vibration in lignin [53–55]. The absorption peak at 1,645 cm^−1^ is attributed principally to a strong interaction between cellulose and absorbed water [56]. The bands around 1,368 cm^−1^ and 1,421 cm^−1^ depicting the asymmetric CH_3_ bending vibration in lignin and the CH_2_ bending deformation in cellulose [55]. The peaks observed between 1,023 cm^−1^ and 1,050 cm^−1^ are responded to C–O–C stretching of cellulose and CH deforming of lignin [55]. Meanwhile, the bands around 898 cm^−1^ are the characteristic peaks of β-(1,4)-glycosidic bond in cellulose [54]. In the present study, the heights of peaks from 898 cm^−1^ to 1,733 cm^−1^ and 2,800 cm^−1^ to 3,400 cm^−1^ showed significant differences after *B. subtilis* RLI2019 treatment (Fig. 8D), and the overall trend of the FTIR spectrum was highly similar to the phenomenon after decomposing wheat straw with *B. subtilis* CD001 [18], illustrating the alteration of chemical functional groups and structural properties in wheat straw. Notably, these findings echo our phenotypic results obtained above, i.e., the contents of detergent fibers, hemicellulose and lignin in wheat straw were drastically lowered after *B. subtilis* RLI2019 degradation (Fig. 7A and B). Taken together, the results of NDF, ADF, hemicellulose, lignin, reducing sugar, FE-SEM, XRD, and FTIR strongly supported that *B. subtilis* RLI2019 can efficiently degrade the lignocellulose components of wheat straw.

## Conclusions

Herein, *B. subtilis* RLI2019 was isolated from the intestine of *R. labralis*. The strain exhibited comprehensive enzymatic activities associated with lignocellulose degradation, including the high activities of endoglucanase, β-glucosidase, xylanase, PASCase, and FPase. Whole genome sequencing demonstrated that *B. subtilis* RLI2019 has probiotic properties and a powerful ability to hydrolyze cellulose and hemicellulose. As a proof of concept, *B. subtilis* RLI2019 was co-cultured with wheat straw for fermentation, which showed that the fiber structure was disrupted and the contents of cellulose, hemicellulose, and lignin were significantly reduced. In summary, *B. subtilis* RLI2019 is an efficient decomposer of lignocellulosic biomass. Furthermore, constructing a complete cellulase system in the strain through genetic engineering is another attractive aspect for future research.

## Materials and methods

### Strains and culture conditions

Cellulolytic strains were previously isolated at different times from goat rumen, yak dung, humus, termite gut, and other environments rich in cellulose-degrading microorganisms, and the strains were stored in our laboratory. For simplicity of description, the strains were sequentially numbered as Isolate C1 ~ C10. In the case of Isolate C6 obtained from the intestine of termites, the isolation and enrichment methods combined serial dilution and Congo red plate screening as described in our previous study [21]. Briefly speaking, the intestine contents of termites were first homogenized and serially diluted in sterile physiological saline solution. The resulting dilutions were plated on Luria–Bertani (LB) agar medium supplemented with sodium carboxymethylcellulose (CMC-Na, Sigma-Aldrich). The plates were then incubated at 37 °C for 24 h, and the size of the hydrolysis zone was determined using Congo red staining [57]. The isolate with the largest hydrolysis zone was further purified and identified based on *16S rRNA* gene sequencing. After the comparative of enzyme activities, Isolate C6 was considered as a dominant cellulolytic bacterium for further study and eventually named as *B. subtilis* RLI2019.

The strains were grown in LB broth (Hopebio, Qingdao, China) or on LB plates (LB medium with 2% agar) at 37 °C unless specifically stated. A 1% (w/v) CMC-Na was dissolved in LB medium to obtain enzyme-producing medium (LC medium). The strains were activated overnight in LB medium; then, the cultures were inoculated to 50 mL LC medium. After culturing for 24 h at 37 °C in an incubator shaker at 220 rpm, the mixture was centrifuged (10,000 rpm) at 4 °C for 15 min to collect the supernatant containing crude enzyme solution. Furthermore, the supernatant was used to evaluate the cellulase activity.

### Assessments of cellulolytic ability

The ability of strains to degrade cellulose was assessed with Congo red staining [57]. Briefly, cellulolytic strains were streaked to form single colonies from frozen glycerol stocks (−80 °C) onto LB plates. The colonies were point-inoculated onto CMC-Na agar plates and continued to grow for 24 h. Subsequently, the plates were incubated in 0.5% (w/v) Congo red solution for 30 min, followed by washing with 1 mol/L NaCl until clear hydrolysis zones were shown. The diameters of the colony (d) and hydrolysis zone (D) around the colony were measured. The ability of cellulose degradation was defined as the ratio of hydrolysis capacity (HCR: D/d).

### Cellulase activity assay

Determining enzyme activity could be influenced by different factors, including the environment for enzyme solution preparation (pH and temperature), methodology, unit definitions, and other variables [8]. Therefore, the cellulase activities were evaluated under the same conditions. Variables can be better controlled so that the results are more rigorous and reliable. Different substrates were prepared as follows: 1% (w/v) CMC-Na for endoglucanase activity; 1% (w/v) xylan from corn cob for xylanase activity; 0.4% (w/v) phosphoric acid swollen cellulose (PASC) for PASCase activity; 5 mM p-nitrophenol glucopyranoside (*pNPG*) for β-glucosidase activity. Additionally, filter paper (Whatman 1#, UK) was divided into pieces (1 cm × 6 cm) as a substrate to determine the enzyme activity of filter paper (FPase). The activities of endoglucanase, PASCase, xylanase, and FPase were determined by the 3,5-dinitrosalicylic acid (DNS) method [9, 58]. The enzyme activity of β-glucosidase was determined by the microdilution method [59].

During all measurement processes, the initial reaction conditions for the enzyme activity assay were set at a consistent incubation temperature (50 °C) and buffer pH (5.5). However, the crude enzyme solution was incubated with the substrate mixture at different times. Specifically, the reaction mixtures contained 2 mL corresponding substrates with 100 μL crude enzyme solution; for the activity assay of endoglucanase, xylanase, PASCase, and FPase, the reaction times were 5, 5, 10, and 30 min, respectively. Besides, 100 µL crude enzyme solution was combined with 100 µL *pNPG*, then incubated at 50 °C for 3 h and terminated by adding 200 μL Na_2_CO_3_ (1 mol/L) to measure β-glucosidase activity. After the termination of reaction, the absorbance values were measured using a multifunctional microplate reader (Bio Tek Instruments, USA). The enzyme unit (U/mL) was expressed as the amount of enzyme required to produce 1 µmol of reducing sugar (glucose, xylose, or p-nitrophenol) per mL/min. Additionally, the effects of culture conditions and reaction conditions on the activities of endoglucanase and xylanase were determined. The maximum value observed for each enzyme was taken as 100%. Relative enzyme activity represents the percentage relative to the highest enzyme activity. All experiments were performed in triplicate.

### Optimization of culture conditions for enzyme production

Different growth times, medium pH, and culture temperatures were evaluated for their effects on endoglucanase and xylanase production. To determine the optimal culture time for the collection of the enzyme solution, the strains were grown in LC medium at 37 °C with shaking for 72 h, and the crude enzyme solution was collected at intervals of 12 h. For the culture temperature profile, the strains were grown in LC medium for 24 h, but the culture temperature was set at 20, 30, 40, and 50 °C, respectively. For the culture pH profile, the crude enzyme solution was collected after 24 h of growth in different LC mediums (pH ranging from 3.5 to 9.5). Standard procedures for enzyme activity assays were performed as described above, and the reaction mixtures were incubated at a temperature of 50 °C and pH 5.5 for 5 min.

### Determination of cell density and total protein concentration

After the culture of *B. subtilis* RLI2019 under different conditions (time, pH, and temperature), the bacterial suspension was collected and diluted appropriately, then the absorbance values at 600 nm were measured as cell density a multifunctional microplate reader (Bio Tek Instruments, USA). Meanwhile, the crude enzyme solution was collected to detect the total protein concentration using Bradford Protein Assay Kit (TIANGEN, China), and the procedures were carried out according to the manufacturer’s instructions.

### Optimization of reaction conditions for enzyme activities

The effects of incubation pH and temperature on endoglucanase and xylanase activities were assessed as a previous procedure [9], with minor modifications. First, enzyme activities on CMC-Na or xylan substrate were determined at a temperature of 50 °C but a buffer pH ranging from 3.5 to 10.5. Furthermore, the enzyme activities were measured at pH 5.5 between 20 °C and 80 °C with increments of 10 °C to determine the optimum temperature for endoglucanase and xylanase activities.

### Thermal stability of endoglucanase and xylanase

The effect of temperature on the stability of endoglucanase and xylanase was measured by incubating the crude enzyme for 1 h at 20, 30, 40, 50, 60, 70, 80, and 90 °C before the addition of the substrate. The reaction mixture was incubated at pH 5.5 (optimal pH) and temperature 60 °C (optimum temperature) for 5 min, then measured reducing sugar and calculated relative enzyme activity as described above.

### Molecular identification of bacterial isolates

The *16S rRNA* gene was amplified using universal bacterial primers 27F (5′-AGAGTTTGATCCTGGCTCAG-3′) and 1492R (5′-GGTTACCTTGTTACGACTT-3′). PCR amplification was performed using a thermal cycler (C1000Touch^™^ system, Bio-Rad, Australia) with high-fidelity PrimeSTAR MAX DNA polymerase (Takara, Japan). The target PCR products were checked by 1% agarose gel electrophoresis and confirmed by Sanger sequencing (Sangon Biotech, Shanghai, China). The sequencing results were submitted to the NCBI Nucleotide BLAST for the identification of bacterial species (http://blast.ncbi.nlm.nih.gov). Furthermore, the *16S rRNA* sequence was submitted to the GTDB database [60], which allowed the top 20 strains to be selected based on the ranking of score values and used to construct the *16S rRNA* phylogenetic tree (Additional file 1: Table S2). Meanwhile, four species closely related to *B. subtilis* (*B. velezensis*, *B. cereus*, *B. thuringiensis*, and *B. altitudini*) as well as three distantly related species (*Escherichia coli*, *Paenibacillus sp.*, and *Virgibacillus sp.*) were included in the phylogenetic tree. The tree was established using MEGA v6.0 software through the neighbor-joining (NJ) method [61].

### Whole genome sequencing, assembly, and annotation

*B. subtilis* RLI2019 was inoculated into LB medium (50 mL), and cultured at 37 °C with shaking (220 rpm) for 24 h. The culture mixture was then centrifuged (9,000 rpm) at 4 °C for 15 min to collect the cell mass, and the supernatant was removed. The cell mass was flash-frozen in liquid nitrogen and used to extract genomic DNA for whole genome sequencing. The whole genome sequencing and assembly were performed by Majorbio Bio-pharm Technology Co., Ltd. (Shanghai, China). First, Illumina Hiseq (100 ×) and PacBio long-read platform (100 ×) were combined to obtain initial raw data. Then, high-quality clean reads were obtained after quality control, which could be used for downstream genome de novo assembly.

Coding DNA sequences (CDSs) were predicted using Glimmer v3.02 [62]. The results of CDSs prediction were analyzed by BLAST against some widely used databases (*E*-value ≤ 1E-5), such as NCBI non-redundant (NR) database, Swiss-Prot database, protein family (Pfam) database, Gene Ontology (GO), Cluster of Orthologous Groups of proteins (COGs) and Kyoto Encyclopedia of Genes and Genomes (KEGGs) [7, 36]. The genome map was drawn using the software of CGView v2.0 [63] or Circos v0.69 [64].

### Carbohydrate-active enzymes (CAZymes) identification

The CAZymes were annotated and classified (E-value ≤ 1E-5) using Diamond v0.8 [65] and hmmscan v3.1 [66] based on the CAZy database (http://www.cazy.org/). According to the similarities in protein sequences, different CAZymes were divided into six protein families, including glycoside hydrolases (GHs), glycosyl transferases (GTs), polysaccharide lyases (PLs), carbohydrate esterases (CEs), carbohydrate-binding modules (CBMs), and auxiliary oxidase activities (AAs).

### Characterization of probiotic sequences

The genome sequence of *B. subtilis* RLI2019 was submitted to the iProbiotics database [35] to predict the probiotic probability of the strain. The frequency distributions of the top 30 significant core features (6-kmer, 7-kmer, and 8-kmer) were counted using Jellyfish software [67].

### Comparative genomic analysis

The genome of *B. subtilis* RLI2019 was compared with other known cellulose-degrading *B. subtilis* strains, including *B. subtilis* TL03, *B. subtilis* 30VD-1, *B. subtilis* Gd7, and *B. subtilis* CRN1 (Table 4). Performed homologous gene analysis using OrthoMCL approach [68].

**Table 4:** The information of comparative genomic of the strains

*B. subtilis* strains	Genome size (bp)	GC content (%)	CDSs	Resources	GenBank accession	References
RLI2019	4,195,306	43.54	4,381	Termite gut	NZ_ CP123621	This study
TLO3	4,232,155	44.10	4,071	Olive tree rhizosphere	GCA_ 002142595	[39]
30VD-1	3,957,760	43.82	3,984	Palm rhizosphere	GCA_ 004366695	[40]
Gd7	4,138,034	43.63	4,375	Decomposing logs	GCA_ 007995205	[41]
CRN1	4,080,381	43.81	4,052	Camel rumen	GCA_ 013267115	[42]

### *B. subtilis* RLI2019 for degradation of wheat straw

To further evaluate the effects of *B. subtilis* RLI2019 to degrade cellulosic biomass. In vitro fermentation experiments of *B. subtilis* RLI2019 co-culture with wheat straw were designed, including wheat straw sterilization pretreatment, strain activation, inoculation, and degradation rate of cellulose determination. The wheat straw samples (10 g) and 50 mL infiltration medium (w/v: 0.5% NH_4_NO_3_, 0.1% MgSO_4_·7H_2_O, and 0.1% NaCl) were thoroughly mixed in the fermentation bags, then the seed solution of *B. subtilis* RLI2019 (5%, v/w) was inoculated into the wheat straw. The mixtures were statically cultured at 37 °C for 7 days, and the bags were fully turned at 24 h intervals to ensure the content of oxygen required for bacterial growth. An equal amount of sterile LB medium was inoculated for the control group in wheat straw. Both treatments were performed in triplicates. After fermentation, the wheat straw samples were oven-dried at 65 °C to constant weight and sifted through a 200-mesh screen for subsequent analyses.

### Determination of detergent fibers, hemicellulose, and lignin

The contents of neutral detergent fiber (NDF) and acid detergent fiber (ADF) were sequentially measured using ANKOM 2000i Fiber Analyzer (ANKOM Technology, Macdon, USA). The contents of hemicellulose and lignin were measured using Hemicellulose Content Assay Kit (Solarbio, China) and Lignin Content Assay Kit (Solarbio, China), respectively. The procedures of sample preparation were performed according to the manufacturer’s instructions.

### Determination of reducing sugars and enzyme activities

The wheat straw fermentation samples (5 g) were mixed with sterile water (20 mL), then the mixtures were incubated at 37 °C for 30 min to obtain the crude extract. Total reducing sugars were determined using the method described by Miller [58]. The activities of endoglucanase and xylanase were assayed as described above. The difference was that the reaction mixtures included crude extract (1 mL), water or substrates (2 mL), and DNS reagent (2.5 mL).

### FE-SEM, XRD, and FTIR analyses

The surface morphological structure of wheat straw was scanned using Field-Emission Scanning Microscope (FE-SEM, Nova Nano SEM-450, USA) to visualize lignocellulose hydrolyzation by *B. subtilis* RLI2019. The cellulose crystallinity index (CrI) of wheat straw was determined using an X-ray diffraction (XRD) instrument (D8 ADVANCE A25, Germany), and the value of CrI was further calculated using the following Segal equation [69]. Fourier transform infrared spectroscopy (FTIR, Vetex70, Germany) was recorded in transmission mode from 4,000 to 400 cm^−1^ with a resolution of 4 cm^−1^, which was further used to evaluate the changes in functional groups.

### Statistical analysis

Statistical analysis was conducted utilizing SPSS v26.0 (Chicago, USA). One-way analysis of variance (ANOVA) was performed to evaluate significance. When a variance of different groups was found, the means were compared via Duncan’s multiple range tests. Student’s t-test was used when appropriate. The results were shown as mean ± standard deviations, and *p*-values below 0.05 were considered statistically significant. GraphPad Prism v8.0 (Bethesda, MD, USA) was used to draw the figures. All genomic data were analyzed on the Majorbio Cloud Platform [70].

### Supplementary Information

**Additional file 1: Figure S1.** Circos genome map of *B. subtilis* RLI2019. **Figure S2.** Venn diagram of homologous genes. **Figure S3.** GO annotation (**A**) and COG annotation (**B**) of the genome sequence. **Table S1.** *16S rRNA* similarity alignment of *B. subtilis* RLI2019 in NCBI database. **Table S2.** *16S rRNA* similarity alignment of *B. subtilis* RLI2019 in GTDB database. **Table S3.** Comparative analyses of top 30 kmer sequences with other *B. subtilis* strains.

**Additional file 2: Table S4.** GO annotation of carbohydrate metabolism genes.

**Additional file 3: Table S5.** COG annotation of carbohydrate metabolism genes.

**Additional file 4: Table S6.** KEGG pathway classification of carbohydrate metabolism genes.

**Additional file 5: Table S7.** CAZyme annotation of the genome sequence.

## Data Availability

The sequencing raw data have been deposited in NCBI SRA database under the Bioproject accession number PRJNA905553. The assembly data can be found at NCBI Genome database under the accession number CP123621. *B. subtilis* RLI2019 was preserved at China Center for Type Culture Collection under the number CCTCC No.M20221488.

## Abbreviations

*B. subtilis*: *Bacillus subtilis*
LB: Luria–Bertani
HCR: Hydrolysis capacity ratio
CMC-Na: Sodium carboxymethylcellulose
PASC: Phosphoric acid swollen cellulose
*pNPG*: *p*-Nitrophenol glucopyranoside
DNS: 3,5-Dinitrosalicylic acid
NCBI: The National Center for Biotechnology Information
CDSs: Coding DNA sequences
GO: Gene Ontology
COGs: Cluster of Orthologous Groups of proteins
KEGGs: Kyoto Encyclopedia of Genes and Genomes
CAZyme: Carbohydrate-active enzyme
GHs: Glycoside hydrolases
GTs: Glycosyl transferases
PLs: Polysaccharide lyases
CEs: Carbohydrate esterases
CBMs: Carbohydrate-binding modules
AAs: Auxiliary activities
NDF: Neutral detergent fiber
ADF: Acid detergent fiber
FE-SEM: Field-emission scanning microscope
CrI: Crystallinity index
XRD: X-ray diffraction
FTIR: Fourier transform infrared spectroscopy
