# Supplementary material for: Whole genome sequencing and the lignocellulose degradation potential of Bacillus subtilis RLI2019 isolated from the intestine of termites
Source: Biotechnol Biofuels Bioprod. 2023 Aug 19;16:130. doi: 10.1186/s13068-023-02375-3 (PMC10439612; doi:10.1186/s13068-023-02375-3)
Supplement: Supplementary file 1 — Additional file 1: Figure S1. Circos genome map of B. subtilis RLI2019. Figure S2. Venn diagram of homologous genes. Figure S3. GO annotation (A) and COG annotation (B) of the genome sequence. Table S1. 16S rRNA similarity alignment of B. subtilis RLI2019 in NCBI database. Table S2. 16S rRNA similarity alignment of B. subtilis RLI2019 in GTDB database. Table S3. Comparative analyses of top 30 kmer sequences with other B. subtilis strains. [file 13068_2023_2375_MOESM1_ESM.docx]

Additional file

Table S1 *16S rRNA* similarity alignment of *B. subtilis* RLI2019 in NCBI database

| Strains | *16S rRNA* accession | Identity % | Align length | Score |
| --- | --- | --- | --- | --- |
| *B. subtilis* kp6 | MH200633.1 | 100.0 | 1442 | 2663 |
| *B. subtilis* ZGL14 | MH362700.1 | 99.93 | 1444 | 2660 |
| *B. subtilis* CICC 10366 | KM365462.1 | 99.93 | 1444 | 2660 |
| *B. subtilis* CS10 | KR780366.1 | 100.0 | 1439 | 2658 |
| *B. subtilis* DP12 | HQ536001.1 | 99.86 | 1446 | 2658 |
| *B. subtilis* CCM9 | HQ536000.1 | 99.86 | 1446 | 2658 |
| *B. subtilis sp.* HY21 | HM579812.1 | 99.93 | 1443 | 2658 |
| *B. subtilis* GD1 | HM055597.1 | 99.86 | 1446 | 2658 |
| *B. subtilis* SU-10 | GU902972.1 | 100.0 | 1439 | 2658 |
| *B. subtilis sp.* 2N-14 | KX214613.1 | 99.93 | 1442 | 2656 |

Table S2 *16S rRNA* similarity alignment of *B. subtilis* RLI2019 in GTDB database

| Strains | GenBank accession | Identity % | Score |
| --- | --- | --- | --- |
| *B. subtilis subsp. subtilis* SRCM101392 | CP021921.1 | 100.0 | 2857 |
| *B. subtilis* HJ0-6 | CP016894.1 | 100.0 | 2857 |
| *B. subtilis subsp. subtilis* KCTC 3135 | CP015375.1 | 100.0 | 2857 |
| *B. subtilis* SG6 | CP009796.1 | 100.0 | 2857 |
| *B. subtilis* BS16045 | CP017112.1 | 99.94 | 2852 |
| *B. subtilis* HJ5 | CP007173.1 | 99.94 | 2852 |
| *B. subtilis* TL03 | CP023257.1 | 99.87 | 2846 |
| *B. subtilis* NCIB 3610 | CP020102.1 | 99.87 | 2846 |
| *B. subtilis* 29R7-12 | CP017763.1 | 99.87 | 2846 |
| *B. subtilis* HRBS-10TDI13 | CP015222.1 | 99.87 | 2846 |
| *B. subtilis* KCTC 1028 | CP011115.1 | 99.87 | 2846 |
| *B. subtilis subsp. subtilis str.* AG1839 | CP008698.1 | 99.87 | 2846 |
| *B. subtilis subsp. subtilis str.* NCIB 3610 | CM000488.1 | 99.87 | 2846 |
| *B. subtilis subsp. subtilis* 6051-HGW | NC_020507.1 | 99.87 | 2846 |
| *B. subtilis subsp. subtilis str.* 168 | NC_000964.3 | 99.87 | 2846 |
| *B. subtilis subsp. subtilis* SRCM100761 | CP021889.1 | 99.81 | 2841 |
| *B. subtilis subsp. spizizenii* TU-B-10 | NC_016047.1 | 99.81 | 2841 |
| *Bacillus sp.* SJ-10 | CP025258.1 | 99.68 | 2830 |
| *B. vallismortis* NBIF-001 | CP020893.1 | 99.68 | 2830 |
| *B. amyloliquefaciens* WS-8 | CP018200.1 | 99.61 | 2824 |

Table S3 Comparative analyses of top 30 kmer sequences with other *B. subtilis* strains

| *B. subtilis* strains  kmer characteristic | | RLI2019 | TLO3 | 30VD-1 | Gd7 | CRN1 |
| --- | --- | --- | --- | --- | --- | --- |
| CGGCAATT | 8-kmer | 131 | 120 | 102 | 131 | 117 |
| AATTGCCG | 8-kmer | 113 | 107 | 107 | 103 | 104 |
| GGCAATTG | 8-kmer | 80 | 75 | 75 | 79 | 68 |
| AATTGCGG | 8-kmer | 81 | 78 | 66 | 74 | 80 |
| ATCATGA | 7-kmer | 636 | 583 | 554 | 618 | 569 |
| CGGAATT | 7-kmer | 407 | 410 | 353 | 394 | 364 |
| AATTGACG | 8-kmer | 104 | 101 | 106 | 101 | 85 |
| CAATTGCC | 8-kmer | 78 | 74 | 67 | 70 | 77 |
| GGTTCA | 6-kmer | 1,099 | 1,056 | 943 | 1,019 | 983 |
| TGAACC | 6-kmer | 1,130 | 1,057 | 1,010 | 1,046 | 1,029 |
| TTCATGA | 7-kmer | 689 | 650 | 610 | 632 | 636 |
| TCATGATC | 8-kmer | 177 | 160 | 139 | 123 | 148 |
| GAATTCA | 7-kmer | 437 | 400 | 374 | 378 | 364 |
| AATGACG | 7-kmer | 399 | 392 | 363 | 371 | 364 |
| GGCAATT | 7-kmer | 307 | 282 | 268 | 305 | 276 |
| TTGTCAGC | 8-kmer | 206 | 203 | 179 | 174 | 192 |
| GCTATGG | 7-kmer | 155 | 147 | 117 | 153 | 131 |
| CCGATTAA | 8-kmer | 127 | 118 | 108 | 115 | 102 |
| CGAATT | 6-kmer | 973 | 930 | 863 | 881 | 872 |
| GATCATGA | 8-kmer | 147 | 136 | 131 | 159 | 138 |
| CGATTAAT | 8-kmer | 89 | 80 | 78 | 72 | 82 |
| TTCATG | 6-kmer | 1,830 | 1,721 | 1,614 | 1,612 | 1,769 |
| AACGGTT | 7-kmer | 453 | 436 | 404 | 398 | 363 |
| CCGATTA | 7-kmer | 361 | 345 | 306 | 341 | 313 |
| GCTCTAT | 7-kmer | 179 | 165 | 144 | 159 | 149 |
| CGTTCAA | 7-kmer | 349 | 338 | 302 | 349 | 352 |
| TTAATCGG | 8-kmer | 107 | 101 | 101 | 100 | 101 |
| AACGGTC | 7-kmer | 232 | 216 | 196 | 202 | 196 |
| CGTCATT | 7-kmer | 406 | 379 | 357 | 374 | 375 |
| GACCGTT | 7-kmer | 215 | 205 | 200 | 208 | 203 |
|  | Total | 11,697 | 11,065 | 10,237 | 10,741 | 10,602 |


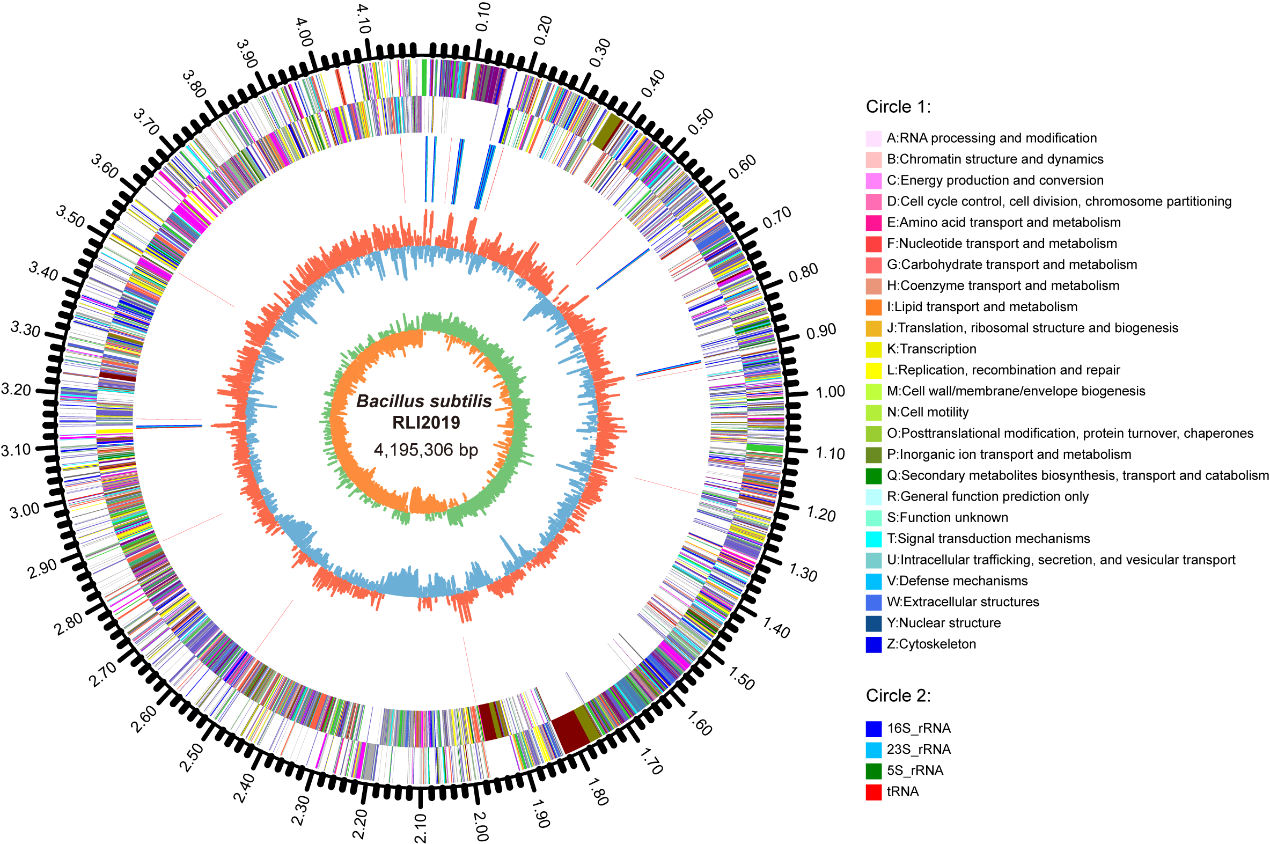


**Fig. S1.** Circos genome map of *B. subtilis* RLI2019. The circles from the inside to the outside are GC contents, rRNA and tRNA, coding sequences on the positive and negative strands, and genome size, respectively.


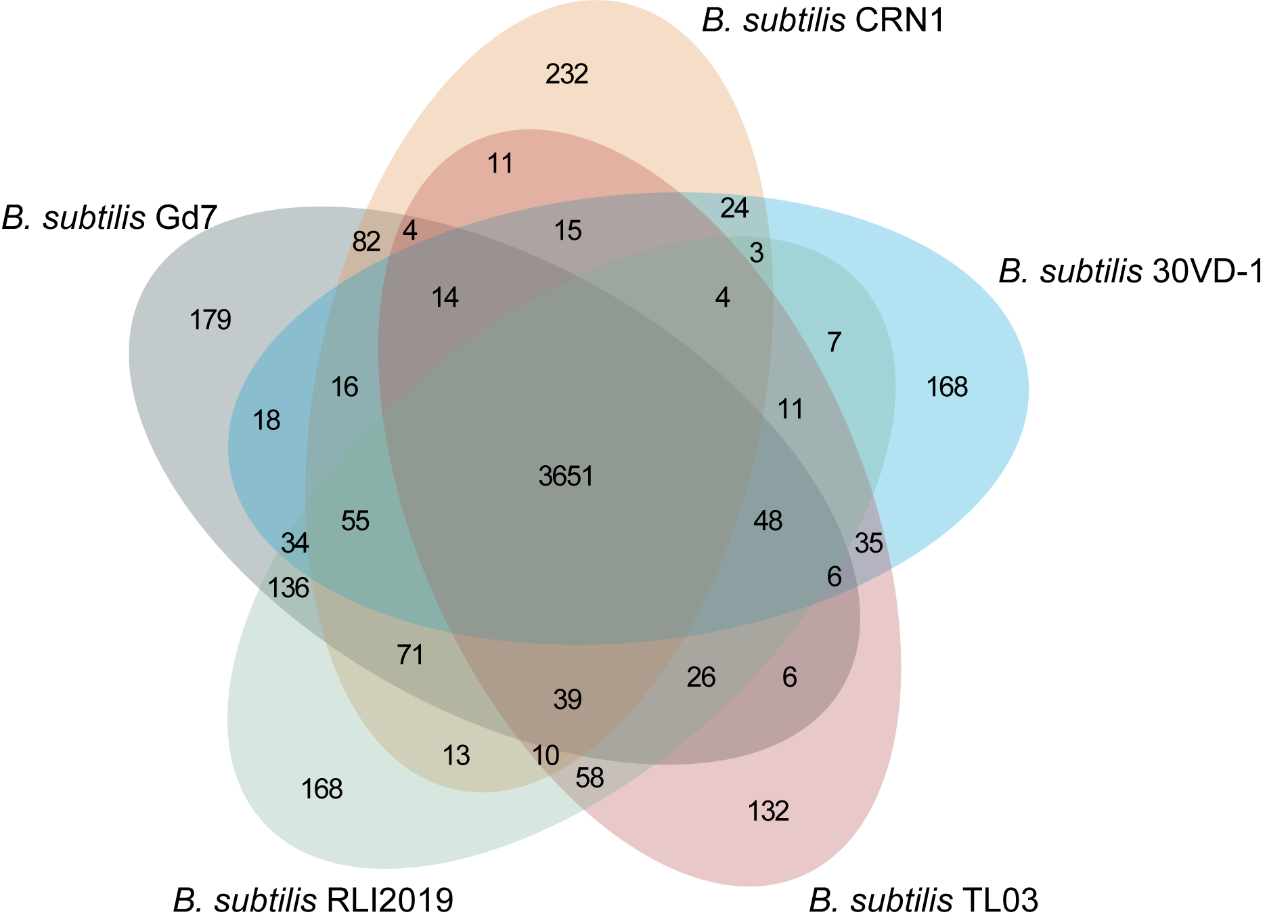


**Fig. S2.** Venn diagram of homologous genes


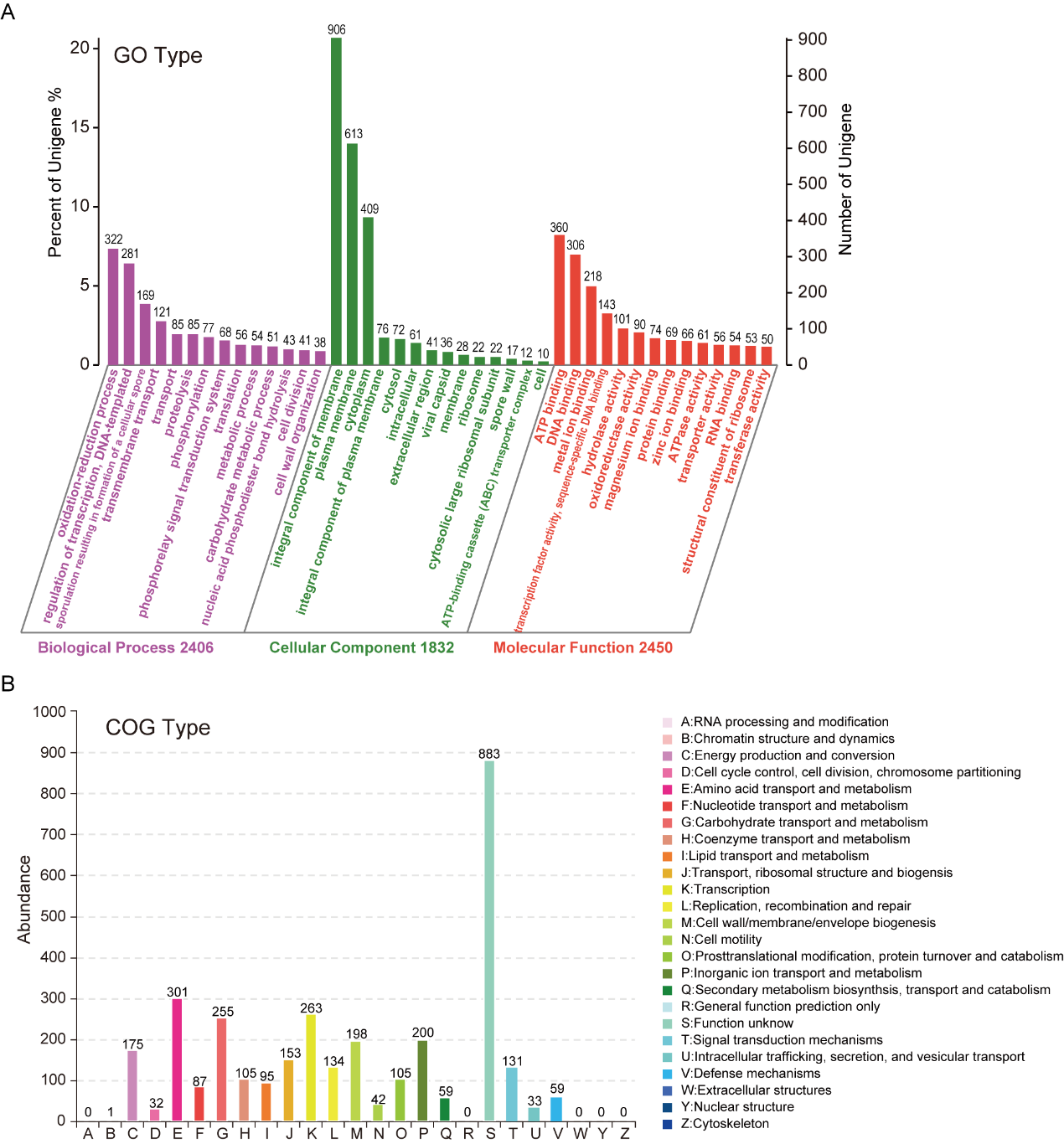


**Fig. S3.** Gene ontology (GO) annotation (**A**) and clusters of orthologous groups of proteins (COGs) annotation (**B**) of the genome sequence.
